# Customised Implant for Temporomandibular Joint: New Technique to Design and Stress Analysis to Balance the Loading at Both Ends

**DOI:** 10.3390/mi14081646

**Published:** 2023-08-20

**Authors:** Anubhav Tiwari, Ishfaq A. Ahmed, Vijay Kumar Gupta, Rakesh Kumar Haldkar, Ivan A. Parinov

**Affiliations:** 1 Machine Dynamics and Vibration Lab, Discipline of Mechanical Engineering, PDPM Indian Institute of Information Technology, Design & Manufacturing, Jabalpur, Dumna Airport Road, Jabalpur 482005, India; 1713605@iiitdmj.ac.in (A.T.); 20mmed07@iiitdmj.ac.in (I.A.A.); vkgupta@iiitdmj.ac.in (V.K.G.); 2 I. I. Vorovich Mathematics, Mechanics and Computer Sciences Institute, Southern Federal University, Rostov-on-Don 344090, Russia; rakeshhaldkar@gmail.com

**Keywords:** customised TMJ implant, DICOM, isotropic, orthotropic

## Abstract

The temporomandibular joint (TMJ) is a critical joint for the opening and closing of the mouth. The generation of customised TMJs according to individuals’ dental anatomy is needed. Currently, the implants available on the market lack consideration of the patient’s dental anatomy. This leads to the creation of an imbalance in the reaction forces on both ends of the TMJ. This requires a slight structural change in the design parameters to give a solution. The purpose of this study is to propose a new design that includes the geometry and materials for a TMJ implant. Stress analysis was carried out on the TMJ to balance the reaction forces at both TMJ ends. A static analysis was performed using ANSYS Workbench, to compare the results of two customised designs of TMJ implants, in order to better balance the reaction forces at both ends. The model in the study showed that the reaction forces for both the patient-specific TMJ implants were nearly balanced. The reaction forces were better balanced, and almost equivalent to the intact conditions. The stresses in the mandible were more uniformly distributed in the customised design of the TMJ implant. The two types of design showed that the custom design took up less space in the patient’s region of surgery, making it a better option compared to a stock TMJ implant. The custom implant would allow faster patient rehabilitation, as the reaction forces would be close to those in intact conditions.

## 1. Introduction

Medical disorders associated with the TMJ, such as ankyloses and osteoarthritis, are commonly discussed in the literature [1]. However, there are several other diseases that affect the regular functioning of the TMJ. Presently, the remedying or treatment of such problems involves physiotherapy, conservative management accompanied by drugs (medication), therapy, splints, arthrocentesis, arthroscopy, or discectomy [2]. In the worst cases, such as bony ankylosis, recurrent fibrous ankylosis, severe degenerative joint disease, aseptic necrosis of the condyle, advanced rheumatoid arthritis, two or more previous TMJ surgeries, the absence of the TMJ structure due to pathology, tumours involving the condyle and mandibular ramus area, or the loss of the condyle due to trauma or pathology, when all the remedies mentioned above are not successful, the replacement of the TMJ by surgery is the only option available to patients [3]. The surgery route for the solution of patient problems related to TMJ is not very straightforward; the success rate of the surgery highly depends on the design of the prosthesis and, at the same time, the process is expensive, too. In order to minimise the possibility of the failure of the prosthesis after surgery, optimisation, in combination with rigorous non-destructive analysis, is essential.

In the past few years, technology in the biomedical domain has grown very rapidly, and the field of the design and development of universal and customised patient-specific implants is gaining a high level of attention from doctors and researchers. However, in the standard model of TMJ prosthesis available on the market, the fossa part is not well fitted [4]. One of the major problems in this area is that everyone has to wait a long time for the output results and, in the meantime, failure can occur, leading to different problems, such as chronic infection, allergy, etc. [5]. On the other hand, custom-made implants are 50% more expensive [6], and require pre-implantation validation. One of the well-known standard TMJ implants is produced by Christensen; it is a metal–metal system in which the condyle and fossa part are connected via a screw arrangement. This Christensen model is now out of production [7].

A comparative study of the TMJ system is rarely available and, therefore, the choice of prosthesis is fully dependent on the experience of the surgeon [8]. A considerable amount of literature is available on the design customisation and non-destructive testing of TMJs using finite element analysis (FEA). One such study was published to compare the custom-made geometry of the fossa component with the standard commercial model [9]. In the study, the load transfer mechanism in the total TMJ model was investigated. In another study, the effect of the screw fixation on a TMJ condylar prosthesis was analysed. In this investigation, the effect of the number of screws on the stress distribution and stability of a TMJ prosthesis was analysed [10]. The conclusion was that only three staggered screws are required to provide the optimum stability for the implant, and the position of these screws significantly affects the strain distribution in the implant [11]. A comparative analysis of different standard TMJ prostheses, made by Christensen Inc. (Richland, Washington, United States) and Techmedica Inc./ TMJ Concepts (California, United States), was carried out, and it was reported that the Techmedica Inc. group had better outcomes compared to the others. Similarly, a detailed review has been presented on the current status of the FEM analysis of TMJ implants [10,11].

Implants are designed to improve patients’ function, and reduce joint disability. According to a survey carried out in 2017, 1000 cases of TMJ reconstruction were observed every year in the USA alone [12]. TMJ Concept and Biomet are two market leaders for TMJ implant manufacturers [13]. While established companies, such as TMJ Concepts and Zimmer Biomet, have a standard design procedure, these implants are designed based on the geometry of a regular American person. At the same time, it is known that the anatomy of Indians is different from that of American people, meaning that it is necessary to include different design considerations for the Indian patient [14]. Currently, there is an urgent need to design and develop implants to meet the needs of Indian patients. In doing so, a few structural parameters need to be changed, so that the problems associated with the earlier design are not carried forwards. To restore the normal working of the TMJ, one needs to generate a structure that is a replica of the standard structure, and then a custom-made TMJ implant can be developed [15]. Custom-made TMJ implants have several drawbacks, such as the need for a precise operation, and an increased lead time for manufacturing [16]. The design of the TMJ implant brings about various adverse effects, such as ear problems, tissue excision, facial nerve dysfunction, infection, allergic reaction, implant wear, or dislocation. The reliability of the implant is key, as, if any failure occurs during regular work, it may lead to problems such as chronic infection or allergy [17], or other problems affecting health. Hence, a brief study and standardised work procedure are needed, to test the reliability. In designing a new implant, one must take care of these adverse effects, and reduce or diminish the adverse effects associated with the TMJ implant. Another substantial side effect observed is stress shielding, in which the density of the bone associated with the implant is reduced, leading to greater porosity and weakness in the bone. To avoid this problem, one needs to create a porous implant, so that proper load transfer occurs. When considering the problems associated with the implant, it is necessary to include the variation in the muscle-loading conditions, from normal conditions (that is, a natural intact mandible) to implant-attached conditions, as, in the implant-attached condition, the body would require time to get used to the rhythm of muscle synchronisation for the opening and closing of the mouth, and mastication changes.

To understand complex structures such as TMJ, one cannot rely on one surgical/medical discipline, but also requires knowledge of multiple areas, such as mechanical and biological material properties, and design.

In this study, a patient-specific TMJ implant was developed. The developed custom-made implant was designed to consider only the condyle region. A static analysis was performed using ANSYS Workbench, and the findings of the simulation study, performed on the custom-designed implants, were analysed. It was observed that the reaction forces for the customised TMJ implants were evenly distributed, and nearly equivalent to the original circumstances, namely the intact mandible condition.

## 2. Modelling

**Figure 1:**
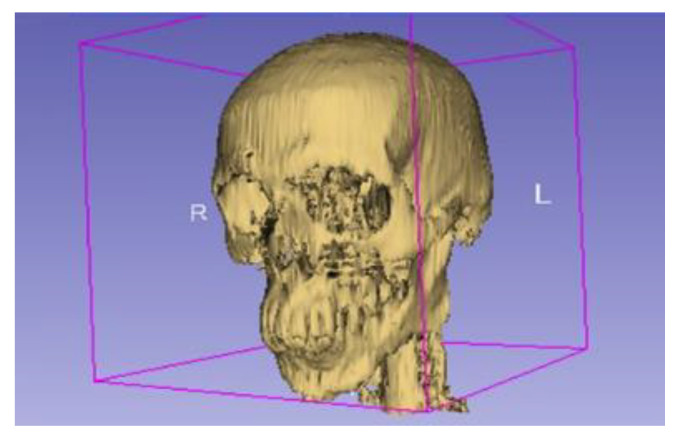
Model illustration generated via 3D Slicer (L-Left, R-Right).

Computed tomography (CT) scan data of a 29-year-old Asian man were gathered. The 3D Slicer imported the DICOM (Digital Image and Communication in Medicine) file obtained in [18]. The three images, namely the axial, sagittal, and coronal planes were visible on the imported data. The axial plane was scanned at a 5 mm offset, resulting in 53 slices, and the coronal and sagittal planes were scanned at a 1 mm offset, resulting in 231 slices on each plane. The 200 HU–1000 HU threshold effect, based on Hounsfield units, was used to distinguish the bone from the surrounding muscle, skin, and tissue. The mandible, skull, and spine models were then separated via the segmenting of each of the slices, in order to create a rough model of the skull and mandible, as depicted in Figure 1.

The model produced via 3D Slicer was not suitable for analysis, as it was in a raw state. Using Meshmixer, smoothening was applied to the surface, to facilitate analysis and create a realistic state. The model was smoothed, before being transferred into CATIA in the shape sculpted as a STL file. The mandible was turned into a solid model once the model had been post-repaired in CATIA for any data lost during conversion [18].

**Figure 2:**
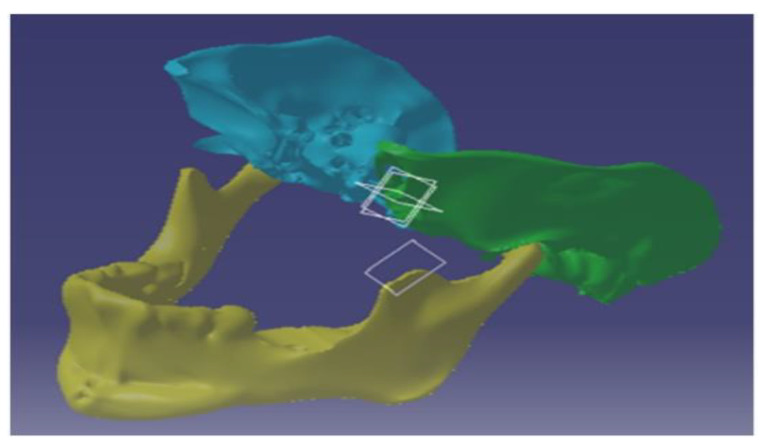
Assembly of the TMJ.

Similarly to the previous phase, the segmented skull model from the DICOM file, generated via 3D Slicer, was imported and smoothed in Meshmixer. Then, a plane cut was made to condense the section of the temporal bone that was of interest. After the plane had been cut, we fixed the model and divided it into the temporal zone. After modelling, the temporal bone was imported into CATIA. The model was transformed to a solid model, with compensation for the decreased thickness in the temporal bone. The assembly of the mandible and temporal bone was performed once both sides of the temporal bone had been sculpted. The distance between the jaw and temporal bones was taken into account when assembling all three models, as depicted in Figure 2.

The sectional gap was taken into account when the assembly was put together, and the articulating disc was modelled on the CATIA workbench and made to touch both sides. The assembly was carried out in a way that ensured the correct fit on the spatial plane, once all three significant components had been modelled. This could be used to simulate and examine stress simulation, and in the implant design.

### Modelling of the Customised Implant and Its Assembly

CT scan data were used to model the implant. The same can also be achieved using the magnetic resonance imaging (MRI) data of the patient. In the current study, the mandible was intact for the available subject. For modelling, the section of the body is cut for which the implant is necessary, and the surface is modelled according to the dimensions of the CT scan data, resulting in an implant model that is identical to the body of the mandible.

**Figure 3:**
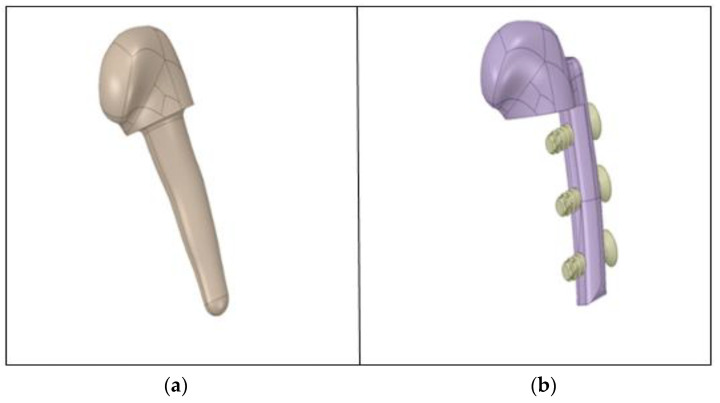
Customised implant for the left side: (**a**) first design with the drilling type, (**b**) second design with a cantilever and external screws (referred to from left to right).

Once this model was generated, the support body was created. In the case of the first design (inspired by dental implants), as shown in Figure 3a, the root is slightly slanted, and has a self-tapping portion at its end. This implant is attached to the mandible via drilling and assembling. The root was made with a slight curve, to increase the contact region, locking the implant and restricting its movement after the operation. In the case of the second design, as shown in Figure 3b, a cantilever protrusion is provided on the implant, such that the surface of the protrusion matches the surface of the mandible body. Furthermore, provision is made to screw the external screw with a slight counter-sink, so that the head of the screw matches the implant surface.

The left side of the mandible was selected for the implant design, because the subject had damaged the left side, and the right side was intact. In the case of a shattered or broken mandible, the surface geometry of the other side will be taken as the guide, and the surface-contact region of the temporal bone will be taken as a reference for the contact surface of the implant, to design the customised implant. Note that the mandible is not perfectly symmetrical, so care should be taken, considering this. One of the essential steps consists of modelling and importing the geometry in the ANSYS^®^ workbench from CATIA. The assembly was performed on the broken mandible and, thereafter, was imported from CATIA to ANSYS^®^ using the STEP file format. The skull part was suppressed, and was not considered for analysis, as it was not the area of interest in the study. Ultra-high-molecular-weight polyethylene (UHMWPE) was used for the glenoid fossa part of the customised TMJ implant.

## 3. Materials and Methods

Four materials were explored for the customised implant. Table 1 [19,20,21] and Table 2 [22,23,24] summarise the material properties. As a result of rigorous literature reviews and the verification of properties that are carcinogenic in humans, Ti alloy, Ti-6Al-4V, Co-Cr-Mo, and ultra-high-molecular-weight polyethylene (UHMWPE) were the materials considered for the implants. The material properties were considered isotropic and orthotropic in nature for the mandible, and were compared. UHMWPE was used for the glenoid fossa part of the customised TMJ implant, and Ti-6Al-4V was considered for the condylar and ramus part. Regarding fasteners, the Ti alloy was used for the screw. This was considered the perfect combination of materials for designing the implant [5].

### Boundary Conditions

The static study was performed considering four conditions:

- Isotropic material property for the mandible with an articular disc.
- Isotropic material property for the mandible without an articular disc.
- Orthotropic material property for the mandible with an articular disc.
- Orthotropic material property for the mandible without an articular disc.

**Figure 4:**
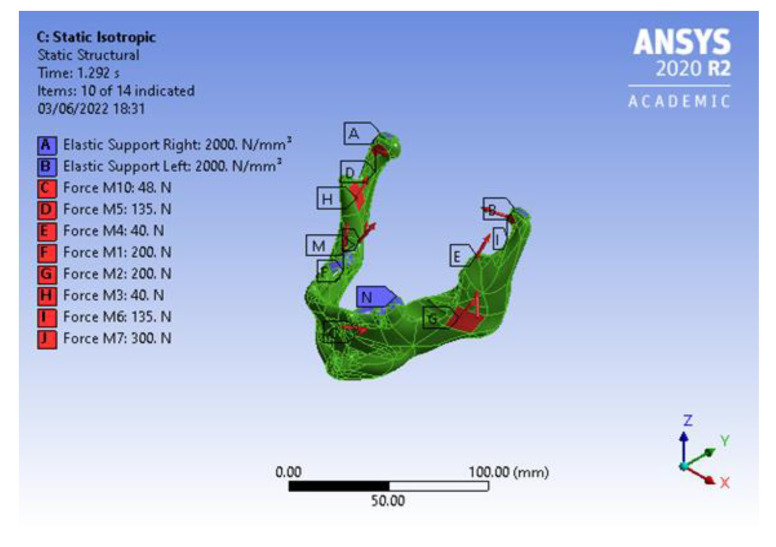
The applied boundary conditions.

In carrying out the study, a bite force of 1000 N was considered, corresponding to maximum chewing forces [25] and the safety factor. The forces are summarised in Table 3 [25]. The forces were applied to the incision points on the body (mandible) and the closure points on the body (skull), as shown in Figure 4. Other boundary conditions are also shown in Figure 4, which include fixed support to the tooth (molar region), because this would be the region of contact of the mandible with the skull, and would not move once the load was applied in the closed-jaw or clenched condition. Elastic support was provided at the region where the articular disc met the skull, because the region would be slightly damped, and would not act as a rigid fixed support.

In this work, a bonded-type connection was used between the customised TMJ implant, mandible, and screw. In the construction of the coordinate system, the centre of gravity of the mandible is considered to be the origin of the coordinate system. The axis is defined as follows:

- The *z*-axis is considered as normal to the axial plane.
- The *y*-axis is considered as normal to the frontal or coronal plane.
- The *x*-axis is considered as normal to the sagittal plane.

**Figure 5:**
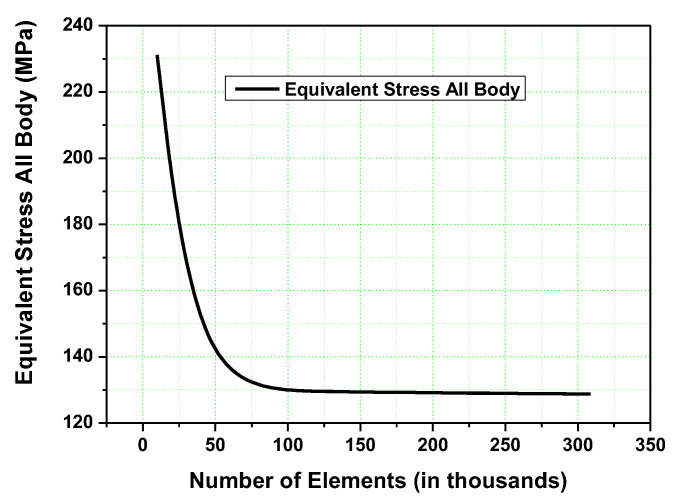
Mesh convergence graph.

In this study, adaptive meshing was performed, using quadratic tetrahedral elements. The intact human mandible was meshed with a varying number of elements, ranging from 5000 to 308,748. For each case, the change in equivalent stress in the whole body was observed. Figure 5 shows the variation in the equivalent stress with the number of elements in the human mandible. With the use of 110,270 elements, a 129.5 MPa pressure was observed. An increase in the number of elements up to 308,748 brings a deviation of the order 0.5%. The equivalent stress converges within 0.5%, and further refinement is not needed.

## 4. Results and Discussions

**Figure 6:**
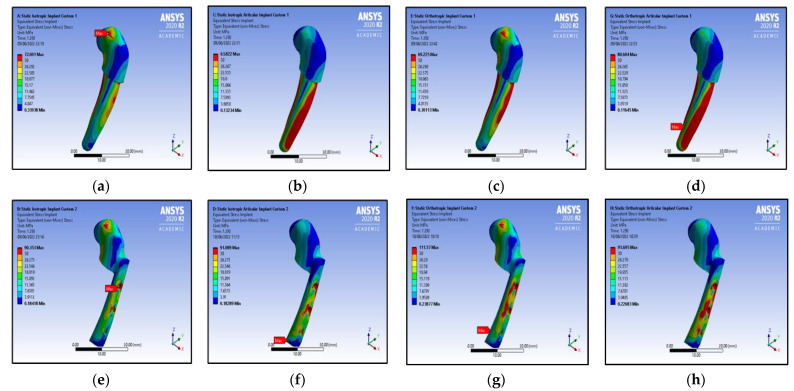
Von Mises stress contours for customised implant-1: (**a**) without an articular disc (isotropic mandible); (**b**) with an articular disc (isotropic mandible); (**c**) without an articular disc (orthotropic mandible); (**d**) with an articular disc (orthotropic mandible); Von Mises stress contours for customised implant-2: (**e**) without an articular disc (isotropic mandible); (**f**) with an articular disc (isotropic mandible); (**g**) without an articular disc (orthotropic mandible); (**h**) with an articular disc (orthotropic mandible).

**Figure 7:**
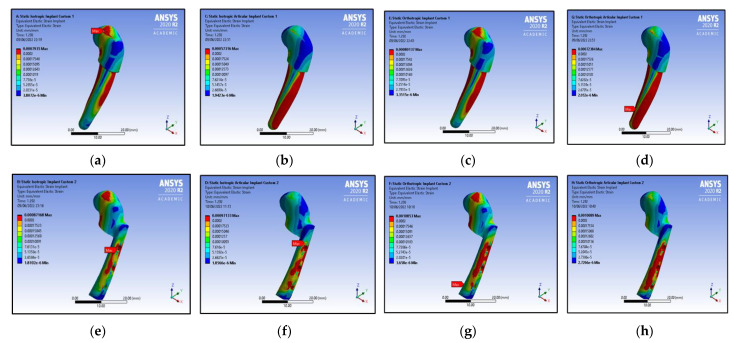
Equivalent strain contours for customised implant-1: (**a**) without an articular disc (isotropic mandible); (**b**) with an articular disc (isotropic mandible); (**c**) without an articular disc (orthotropic mandible); (**d**) with an articular disc (orthotropic mandible); Equivalent strain contours for customised implant-2: (**e**) without an articular disc (isotropic mandible); (**f**) with an articular disc (isotropic mandible); (**g**) without an articular disc (orthotropic mandible); (**h**) with an articular disc (orthotropic mandible).

The simulation study of the customised implant and intact mandible were performed with ANSYS^®^ software_._ For the implanted and intact mandible results of all four studies, namely (i) the isotropic mandible with an articular disc, (ii) the isotropic mandible without an articular disc, (iii) the orthotropic mandible with an articular disc, and (iv) the orthotropic mandible without an articular disc, are summarised and depicted in Figure 6 and Figure 7 and in Table 4, Table 5 and Table 6.

From Figure 6a,b, it was observed that, in the case of Customised TMJ Implant 1, the value of von Mises stress for the isotropic mandible without an articular disc property is higher than the value for the isotropic mandible with an articular disc property, whereas the value of von Mises stress for the orthotropic mandible without an articular disc property is lower than the value for the orthotropic mandible with an articular disc property, as depicted in Figure 6c,d.

As shown in Figure 6e,f, the von Mises stress value for the isotropic mandible without an articular disc property, in the case of Customised TMJ Implant 2, is lower than the value for the isotropic mandible with an articular disc property. A similar trend was observed for the screws in Customised Implant 2.

In accordance with Figure 6g,h, the von Mises stress value for the orthotropic mandible without an articular disc property is higher than the value for the orthotropic mandible with an articular disc property in the case of Customised TMJ Implant 2. However, in the case of the screws in Customised TMJ Implant 2, the von Mises stress value for the orthotropic mandible without an articular disc property is lower than the value for the orthotropic mandible with an articular disc property.

The equivalent strain value for the isotropic mandible without an articular disc property is higher than the value for the isotropic mandible with an articular disc property in the case of Customised TMJ Implant 1, as shown in Figure 7a,b. Similarly, the equivalent strain value for the orthotropic mandible without an articular disc property is slightly higher than the value for the orthotropic mandible with an articular disc property, as shown in Figure 7c,d.

As shown in Figure 7e,f, the equivalent strain value for the isotropic mandible without an articular disc property in the case of Customised TMJ Implant 2 is lower than the value for the isotropic mandible with an articular disc property. A similar trend was observed for the screws in Customised Implant 2.

Further, as shown in Figure 7g,h, the equivalent strain value for the orthotropic mandible without an articular disc property is slightly higher than the value for the orthotropic mandible with an articular disc property, in the case of Customised TMJ Implant 2. However, in the case of the screws in Customised TMJ Implant 2, the equivalent strain value for the orthotropic mandible without an articular disc property is lower than the value for the orthotropic mandible with an articular disc property.

From Table 5, it can also be observed that the summation of the force vector in both the fixed support region and the elastic support is equal to 1000 N, satisfying the required condition and, thus, verifying the model. Verification consists of cross-checking the results obtained with the input boundary conditions; in this research study, force is the primary input function given to the body. As the reaction force, obtained from the results, is equal to the input force, the results obtained are correct. It is observed that the strain value in the body is higher for orthotropic material, compared to isotropic material. This is because the orthotropic material property provides directional strength. However, it is observed that the body as a whole experiences less stress. Additionally, the maximum stress value is decreased (with one exception). The difference in stress levels is greater for the highest stress of 17% (exception in one instance, where it is −6.8%), than for the average stress of 1–3% throughout the body as a whole.

**Figure 8:**
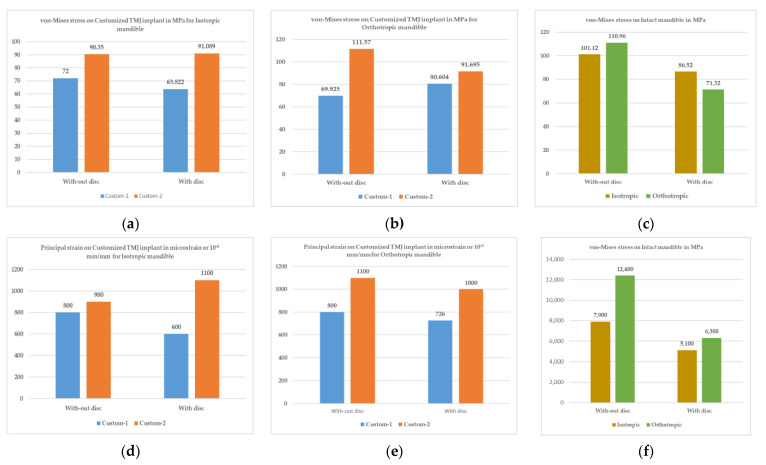
Comparison of Custom 1 and Custom 2 TMJ implants: (**a**) von Mises stress for the isotropic material, (**b**) von Mises stress for the orthotropic material, (**c**) von Mises stress for the intact mandible, (**d**) principal strain for the isotropic material, (**e**) principal strain for the orthotropic material, (**f**) principal strain for the intact mandible.

From Table 4 and Table 5 and Figure 8, it can be observed that the properties of the orthotropic material offer a more distributed loading condition, and a higher stress value, than the properties of the isotropic material. Further, from Table 5, it can be observed that Customised Implant 2 is most affected, and Figure 6 represents the location of the implant which is most affected in the case of static study. On the other hand, custom design 1 has uniformly distributed stress. The reason for the more stress-affected body in custom design 2 is the improper surface-to-surface contact, and the presences of the screw thread. Custom design 1 has a more uniform stress distribution, due to a good surface-to-surface contact and an even distribution of the stress throughout the body. This is helpful, because it will promote calcium formation in the bone, and increase its strength. It can be observed that the most critical part is the balance in the reaction force on both sides, which is shown in Table 4. The custom design achieves this properly, because of the customisation of the implant. It is also observed that the difference in the reaction force is in the range of 0 to 8% in the case of Custom Implant 1 and Custom Implant 2 for the static case.

The results of the static study for the intact mandible are summarised in Table 6, and shown in Figure 8c,f. It was observed that the articular disc was one of the essential bodies to absorb the force distribution near the contact region, and deformed to a greater extent to withstand the loading conditions. From the comparison study, it was observed that the articular disc shifted the load towards the condylar head, minimising the stress impact on the mandible body. In contrast, the deformation in the disc region increased, due to its lower Young’s modulus. The orthotropic material properties distributed the stress throughout the body, and demonstrated its benefits. It was also observed that the deformation in the study considering the articular disc property was too high, near to 0.7 mm in the isotropic case and 0.96 mm in the case of the orthotropic material. Thus, by adding the articular disc, the material became elastic (a hyperelastic material), and hence deforms more. So, adding the articular disc property into the model makes the model safer for the mandible body, by distributing stresses throughout the body.

From the study, it was also observed that the maximum stress in the case of Customised Implant 1 was less than that in Customised Implant 2. However, these data cannot be generalised because the stress value might be localised within specific areas only. As can be seen from the result for Customised Implant 2, the maximum observed stress is in the area of the screw drilled into the mandible. So, the characteristic that would give a better result is the average stress across the body.

The average stress value for the two cases of customised TMJ implants is less than the intact mandible condition, showing that stress shielding would occur in all conditions. However, Table 5 clearly shows that Customised Implant 1 shares and distributes the masticating load (bite force) to the bone tissue (the human mandible) better than Customised Implant 2. This implies that customised TMJ Implant 2 is more prone to stress shielding than the design of Customised Implant 1 is.

From the observed results, it can be stated Customised Implant 1 is better. The issue which may arise with Customised Implant 1 touches on its assembly within the patient, because there is not much space in the region for surgery, and the implant requires drilling into the axial plane.

It was observed that the articular disc is one of the essential bodies to absorb the force distribution near the contact region. It deforms to a greater extent to withstand the loading condition. The maximum stress is reduced from 47 to 26% in the study case, with the articular disc property in all bodies, while, in the mandible body alone, it is reduced from 36 to 8%. Thus, it can be stated that the articular disc helps in the absorbing and shifting of load towards the condylar head, and minimises the stress impact on the mandible body. The customised implant design shows a better distribution of stress throughout the body. The reaction forces obtained from the customised implant are more balanced, thus making the design more user-friendly, and allowing the patient more relaxation after the operation, and an easier recovery. The stress-shielding process is reduced, providing a better solution than the currently available implants.

## 5. Conclusions

This research study presents an approach to the modelling of the temporomandibular joint region using the patient’s CT scans. The research work discusses the steps for modelling the TMJ, and its analysis in static modes. The analysis can help in the design and development of patient-specific implants. The model developed could be used to plan surgeries and practice surgery. This can be achieved by creating a digital model or digital twin, comprehending the areas where incisions are made during the operation, and accurately determining the stress levels in the temporomandibular region, locating the crucial facial nerves. On the basis of the results obtained, it can be stated that the orthotropic material results in a better stress distribution, compared to the isotropic material. The orthotropic material has a greater impact on the temporomandibular modeling than the isotropic material, as most of the orthotropic cases exhibit higher von Mises stresses, in general.

The current study is based on the material properties obtained through the range of literature; the actual material properties may differ. Further, only isotropic and orthotropic materials are considered. In actual practice, material shows great variation, and this can be explored in future. The experimental validation of the results can be carried out in the near future.

Further, the design of the implant affects the stress shielding. Custom design 1 prevents the stress-shielding effect better than custom design 2 (as shown in Table 5), and may be preferred as a suitable customised TMJ implant. Based on the current study, it can be concluded that prior modelling and analysis of the customised TMJ implant may result in better the functioning and performance of the human TMJ implant.

## Figures and Tables

**Table 1:** Mechanical properties of the materials used for the customised implant [19,20,21].

Material Properties	Co-28Cr-6Mo	Ti-6Al-4V	Ti-Alloy	UHMWPE	Articular Disc
Density (kg/m^3^)	8300	4429	4620	940	1134
Young’s modulus (MPa)	210,000	113,800	96,000	928	44.1
Poisson’s ratio	0.2999	0.3387	0.36	0.4216	0.4

**Table 2:** Mechanical properties used for the human mandible [22,23,24].

Property Bone Type	Value
**Cortical Bone** **(Orthotropic property)**	
Density (kg/m^3^)	1134
Young’s modulus, *x*-direction (MPa)	10,800
Young’s modulus, *y*-direction (MPa)	19,400
Young’s modulus, *z*-direction (MPa)	13,300
Poisson’s ratio, *xy*-plane	0.249
Poisson’s ratio, *yz*-plane	0.224
Poisson’s ratio, *xz*-plane	0.309
**Cortical Bone** **(Isotropic property)**	
Young’s modulus (MPa)	19,000
Poisson’s ratio	0.3

**Table 3:** Muscle forces [25].

Muscle Name	Nom.	Force (N)
Right masseter	M1	200
Left masseter	M2	200
Right temporalis	M3	40
Left temporalis	M4	40
Right lat. pterygoid	M5	135
Left lat. pterygoid	M6	135
Right med. Pterygoid	M7	300
Left med. pterygoid	M8	300
Right ant. digastric	M9	48
Left ant. digastric	M10	48

**Table 4:** Results for all the studies on all the bodies in two customised TMJ implant types (static study).

Implant Type	Mandible Material Property	Considering the Articular Disc Property	Stress on All Bodies		Strain on All Bodies		Deformation in μm		Reaction Force	
			Max. (MPa)	Avg. (MPa)	Max.	Avg.	Max.	Avg.	Right (N)	Left (N)
Mandible intact condition	Isotropic	Without	127.7	8.5	10.2 × 10^−3^	0.4 × 10^−3^	150	60	332	326
	Isotropic	With	86.5	10.8	982.4 × 10^−3^	5.7 × 10^−3^	700	390	686	713
	Orthotropic	Without	136.4	8.4	15.4 × 10^−3^	0.6 × 10^−3^	230	80	322	321
	Orthotropic	With	71.3	10.5	1241 × 10^−3^	7.6 × 10^−3^	960	550	640	655
Custom type 1	Isotropic	Without	96.2	9.4	6 × 10^−3^	0.3 × 10^−3^	140	60	345	315
	Isotropic	With	81.9	11.4	830 × 10^−3^	6.4 × 10^−3^	641	420	688	705
	Orthotropic	Without	97.4	10.0	8 × 10^−3^	0.3 × 10^−3^	210	73	337	308
	Orthotropic	With	80.6	11.8	1049 × 10^−3^	8 × 10^−3^	902	580	644	644
Custom type 2	Isotropic	Without	119.9	8.1	3 × 10^−3^	0.2 × 10^−3^	140	62	341	314
	Isotropic	With	156.4	7.9	320 × 10^−3^	2 × 10^−3^	620	390	686	701
	Orthotropic	Without	145.7	9.6	4.5 × 10^−3^	0.2 × 10^−3^	220	79	331	307
	Orthotropic	With	203.8	8.7	370 × 10^−3^	2.7 × 10^−3^	890	530	641	641

**Table 5:** Results for all four studies per body and the two customised TMJ implant types (static study).

Implant Type	Mandible Material Property	Considering the Articular Disc Property	Stress on Mandible (MPa)		Strain on Mandible		Stress on Implant (MPa)		Strain on Implant		Stress on Screws (MPa)		Strain on Screws	
			Max.	Min.	Max.	Min.	Max.	Min.	Max.	Min.	Max.	Min.	Max.	Min.
	Isotropic	Without	56	5.5	0.3 × 10^−3^	0.3 × 10^−3^	72	16.8	0.8 × 10^−3^	0.1 × 10^−3^	-	-	-	-
Custom type 1	Isotropic	With	82	8.0	5.2 × 10^−3^	0.4 × 10^−3^	64	20.8	0.6 × 10^−3^	0.2 × 10^−3^	-	-	-	-
	Orthotropic	Without	57	4.8	4.4 × 10^−3^	0.4 × 10^−3^	69	20.0	0.8 × 10^−3^	0.2 × 10^−3^	-	-	-	-
	Orthotropic	With	72	6.7	6.3 × 10^−3^	0.5 × 10^−3^	81	24.6	1.0 × 10^−3^	0.2 × 10^−3^	-	-	-	-
	Isotropic	Without	50	4.9	2.8 × 10^−3^	0.3 × 10^−3^	90	11.3	0.9 × 10^−3^	0.1 × 10^−3^	120	8.5	1.5 × 10^−3^	0.1 × 10^−3^
Custom type 2	Isotropic	With	116	5.5	7.2 × 10^−3^	0.3 × 10^−3^	91	11.2	1.0 × 10^−3^	0.1 × 10^−3^	156	10.3	1.7 × 10^−3^	0.1 × 10^−3^
	Orthotropic	Without	50	5.1	4.5 × 10^−3^	0.4 × 10^−3^	112	13.9	1.1 × 10^−3^	0.1 × 10^−3^	146	10.3	1.8 × 10^−3^	0.1 × 10^−3^
	Orthotropic	With	127	5.4	9.5 × 10^−3^	0.4 × 10^−3^	92	12.0	1.0 × 10^−3^	0.1 × 10^−3^	204	12.1	2.2 × 10^−3^	0.1 × 10^−3^

**Table 6:** Von Mises stress and strain for the intact mandible with four studies per body (static study).

Mandible Material Property	Considering Articular Disc Property	Stress on Mandible		Strain on Mandible		Stress on Articular Disc		Strain on Articular Disc	
		Max. (MPa)	Avg. (MPa)	Max.	Avg.	Max. (MPa)	Avg. (MPa)	Max.	Avg.
Isotropic	Without	101.12	8.3727	0.0079	0.0004	127.73	9.4831	0.0102	0.0005
Isotropic	With	86.52	11.6540	0.0051	0.0006	28.12	2.4620	0.9824	0.0574
Orthotropic	Without	110.96	8.2524	0.0124	0.0006	136.44	9.4982	0.0154	0.0008
Orthotropic	With	71.32	11.2630	0.0063	0.0008	35.66	3.2209	1.2405	0.0751

## Data Availability

The CT scan data is not publicly available due to ethical restrictions.

## References

[1] Abel E.W., Hilgers A., Mcloughlin P.M. (2015). Finite element analysis of a condylar support prosthesis to replace the temporomandibular joint. Br. J. Oral Maxillofac. Surg..

[2] Chase D.C., Hudson J.W., Gerard D.A., Russell R., Chambers K., Curry J.R., Latta J.E., Christensen R.W. (1995). The Christensen prosthesis: A retrospective clinical study. Oral Surg. Oral Med. Oral Pathol..

[3] Mercuri L.G., Ali F.A., Woolson R. (2008). Outcomes of total alloplastic replacement with periarticular autogenous fat grafting for management of reankylosis of the temporomandibular joint. J. Oral Maxillofac. Surg..

[4] Sagl B., Schmid-Schwap M., Piehslinger E., Kundi M., Stavness I. (2022). Effect of facet inclination and location on TMJ loading during bruxism: An in-silico study. J. Adv. Res..

[5] Aagaard E., Thygesen T. (2014). A prospective, single-centre study on patient outcomes following temporomandibular joint replacement using a custom-made Biomet TMJ prosthesis. Int. J. Oral Maxillofac. Surg..

[6] TMJ Dysfunction. https://tmjreliefclinic.com.au/tmj-dysfunction-or-tmd.

[7] Koolstra J.H., Van Eijden T.M.G.J. (1995). Biomechanical analysis of jaw-closing movements. J. Dent. Res..

[8] Tiwari A., Gupta V.K., Haldkar R.K., Parinov I.A. (2022). Biomechanical Analysis of patient-specific temporomandibular joint implant and comparison with natural intact jaw bone using finite element method. Appl. Sci..

[9] Hannam A.G., Stavness I., Lloyd J.E., Fels S. (2008). A dynamic model of jaw and hyoid biomechanics during chewing. J. Biomech..

[10] What Is TMJ (or TMD)?. https://www.webmd.com/oral-health/ss/slideshow-tmj-tmd-overview.

[11] TMJ.com. http://www.tmj.com/disorder/.

[12] Onoriobe U., Miloro M., Sukotjo C., Mercuri L.G., Lotesto A., Eke R. (2016). How Many Temporomandibular Joint Total Joint Alloplastic Implants Will Be Placed in the United States in 2030?. Int. J. Oral Maxillofac. Surg..

[13] Driemel O., Braun S., Müller-Richter U., Behr M., Reichert T., Kunkel M., Reich R. (2009). Historical development of alloplastic temporomandibular joint replacement after 1945 and state of the art. Int. J. Oral Maxillofac. Surg..

[14] Shan X.F., Chen H.M., Liang J., Huang J.W., Cai Z.G. (2015). Surgical reconstruction of maxillary and mandibular defects using a printed titanium mesh. J. Oral Maxillofac. Surg..

[15] Ackland D.C., Robinson D., Redhead M., Lee P.V.S., Moskaljuk A., Dimitroulis G. (2017). A personalized 3D-printed prosthetic joint replacement for the human temporomandibular joint: From implant design to implantation. J. Mech. Behav. Biomed. Mater..

[16] Mańkowski J., Piękoś J., Dominiak K., Klukowski P., Fotek M., Zawisza M., Żach P. (2021). A Mandible with the Temporomandibular Joint—A new FEM model dedicated to strength and fatigue calculations of bonding elements used in fracture and defect surgery. Materials.

[17] Festa F., Galluccio G. (1998). Clinical and experimental study of TMJ distraction: Preliminary results. Cranio.

[18] Ahmed I.A., Gupta V.K., Tiwari A. Modelling of temporomandibular joint. Proceedings of the CAD’22.

[19] American Elements. https://www.americanelements.com/cobalt-chromium-molybdenum-alloy-105525-46-0.

[20] Rodrigues Y.L., Mathew M.T., Mercuri L.G., da Silva J.S.P., Henriques B., Souza J.C.M. (2018). Biomechanical simulation of temporomandibular joint replacement (TMJR) devices: A scoping review of the finite element method. Int. J. Oral Maxillofac. Surg..

[21] Ramos A., Mesnard M. (2015). Christensen vs Biomet Microfixation alloplastic TMJ implant: Are there improvements? A numerical study. J. Cranio-Maxillo-Facial Surg..

[22] Ashman R., Van Buskirk W. (1987). The elastic properties of a human mandible. Adv. Dent. Res..

[23] Meema H.E., Meema S. (1978). Compact bone mineral density of the normal human radius, Acta Radiologica: Oncology, Radiation, Physics. Biology.

[24] Tanaka E., Rodrigo D.P., Tanaka M., Kawaguchi A., Shibazaki T., Tanne K. (2001). Stress analysis in the TMJ during jaw opening by use of a three-dimensional finite element model based on magnetic resonance images. Int. J. Oral Maxill.

[25] Korioth T.W.P., Hannam A.G. (1994). Deformation of the human mandible during simulated tooth clenching. J. Dent. Res..

